# Supplementary material for: MetaQTL: a package of new computational methods for the meta-analysis of QTL mapping experiments
Source: BMC Bioinformatics. 2007 Feb 8;8:49. doi: 10.1186/1471-2105-8-49 (PMC1808479; doi:10.1186/1471-2105-8-49)
Supplement: Additional File 7 — MetaQTL Package : jar file and tutorial. This Zip archive contains both the MetaQTL JAR file and the files of the tutorial. [file 1471-2105-8-49-S7.zip › org.inra.metaqtl/doc/org/metaqtl/algo/QtlProjAlgorithm.html]

QtlProjAlgorithm


|  |  |  |
| --- | --- | --- |
| **Overview** \| **Package** \| **Class** \| **Use** \| **Tree** \| **Deprecated** \| **Index** \| **Help** | |  |
| **PREV CLASS**   **NEXT CLASS** | **FRAMES**    **NO FRAMES**     **All Classes** |  |
| SUMMARY: NESTED \| FIELD \| CONSTR \| METHOD | DETAIL: FIELD \| CONSTR \| METHOD |  |


---


## org.metaqtl.algo Class QtlProjAlgorithm

```
java.lang.Object
  org.metaqtl.algo.MetaAlgorithm
      org.metaqtl.algo.QtlProjAlgorithm
```

**All Implemented Interfaces:**: IMetaAlgorithm

---

``` public class QtlProjAlgorithm extends MetaAlgorithm ```

This class defines some methods to project QTL positions from
a genetic map to another.

---

| **Field Summary** | |
| --- | --- |
| `int` | `sd_mode`             The mode of compuation of the standard deviation of the QTL positions. |

**Fields inherited from class org.metaqtl.algo.MetaAlgorithm**

`logger, loggerUp, workAmount, workProgress`


**Constructor Summary**

`QtlProjAlgorithm(IBioGenome[] maps,
IBioGenome skeleton,
IBioLocus[] dubious)`
  
          Creates a new instance of `QtlProjAlgorithm` for
the given configuration of projection.


| **Method Summary** | |
| --- | --- |
| `void` | `checkProjection(ChromCluster[] chromClusters)` |
| `void` | `doProjection(ChromCluster[] chromClusters)` |
| `Chromosome[]` | `getResult()` |
| `void` | `run()`             This routine performs the projection of the QTL from the maps onto the skeleton and returns the chromosomes on which the projection has been done. |
| `void` | `setPvalue(double p)` |
| `void` | `setRatio(double r)` |

**Methods inherited from class org.metaqtl.algo.MetaAlgorithm**

`disableLogger, getLogger, getWorkAmount, getWorkProgress, isLoggerEnable, setLogger, setLogger`

**Methods inherited from class java.lang.Object**

`clone, equals, finalize, getClass, hashCode, notify, notifyAll, toString, wait, wait, wait`

**Field Detail**

### sd\_mode

```
public int sd_mode
```

:   The mode of compuation of
    the standard deviation of the QTL positions.


**Constructor Detail**

### QtlProjAlgorithm

```
public QtlProjAlgorithm(IBioGenome[] maps,
                        IBioGenome skeleton,
                        IBioLocus[] dubious)
```

:   Creates a new instance of `QtlProjAlgorithm` for
    the given configuration of projection.

    **Parameters:**: `maps` - the maps from which the Qtl would to be projected.: `skeleton` - the map on which the Qtl would be projected.: `dubious` - an optional list of dubious marker which would not be taken into account in the projection.


**Method Detail**

### getResult

```
public Chromosome[] getResult()
```

:   **Returns:**

---


### run

```
public void run()
```

:   This routine performs the projection of the QTL from the maps onto the
    skeleton and returns the chromosomes on which the projection has been
    done.

---


### checkProjection

```
public void checkProjection(ChromCluster[] chromClusters)
```

:   **Parameters:**: `chromClusters` -

---


### doProjection

```
public void doProjection(ChromCluster[] chromClusters)
```

:   **Parameters:**: `chromClusters` -

---


### setRatio

```
public void setRatio(double r)
```

:   **Parameters:**: `d` -

---


### setPvalue

```
public void setPvalue(double p)
```


---


|  |  |  |
| --- | --- | --- |
| **Overview** \| **Package** \| **Class** \| **Use** \| **Tree** \| **Deprecated** \| **Index** \| **Help** | |  |
| **PREV CLASS**   **NEXT CLASS** | **FRAMES**    **NO FRAMES**     **All Classes** |  |
| SUMMARY: NESTED \| FIELD \| CONSTR \| METHOD | DETAIL: FIELD \| CONSTR \| METHOD |  |


---
